# Supplementary figures and images for: Inner hair cell dysfunction in Klhl18 mutant mice leads to low frequency progressive hearing loss
Source: PLoS One. 2021 Oct 1;16(10):e0258158. doi: 10.1371/journal.pone.0258158 (PMC8486144; doi:10.1371/journal.pone.0258158)

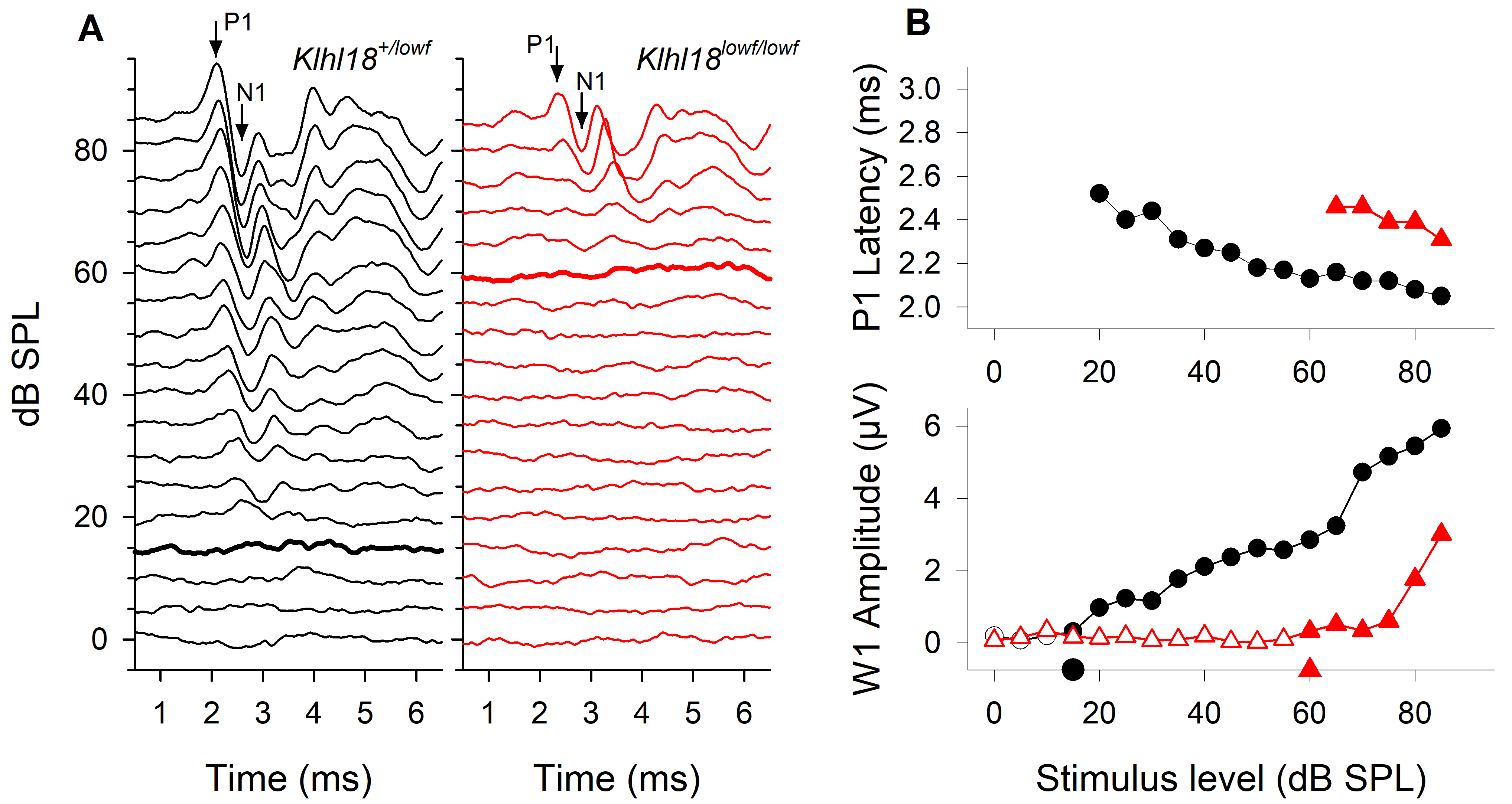

Supplement: S1 Fig — A, Representative ABRs recorded in response to 12 kHz tone pips presented from 0–85 dB SPL are plotted for a Klhl18+/lowf control mouse (black) and a Klhl18lowf/lowf mutant mouse (red). P1 and N1 indicate the positions of the first positive and negative peaks of the waveform, from which amplitude and latency measurements are make. The thickened line on each stack of ABR traces indicates the visually-determined threshold level for these responses (15 dB SPL for the control mouse; 60 dB SPL for the mutant mouse). B, Input-Output functions for ABR wave 1 amplitude and P1 latency measurements (from the traces shown in A) are plotted as a function of stimulus sound pressure level (dB SPL), for the control mouse (black circles) and mutant mouse (red up-triangles). Sub-threshold points are indicated by open symbols. Supra-threshold points are indicated by filled symbols. The estimated threshold for each mouse is indicated by the appropriate single symbol on the abscissa of the wave 1 amplitude function. (TIF) [file pone.0258158.s001.tif]

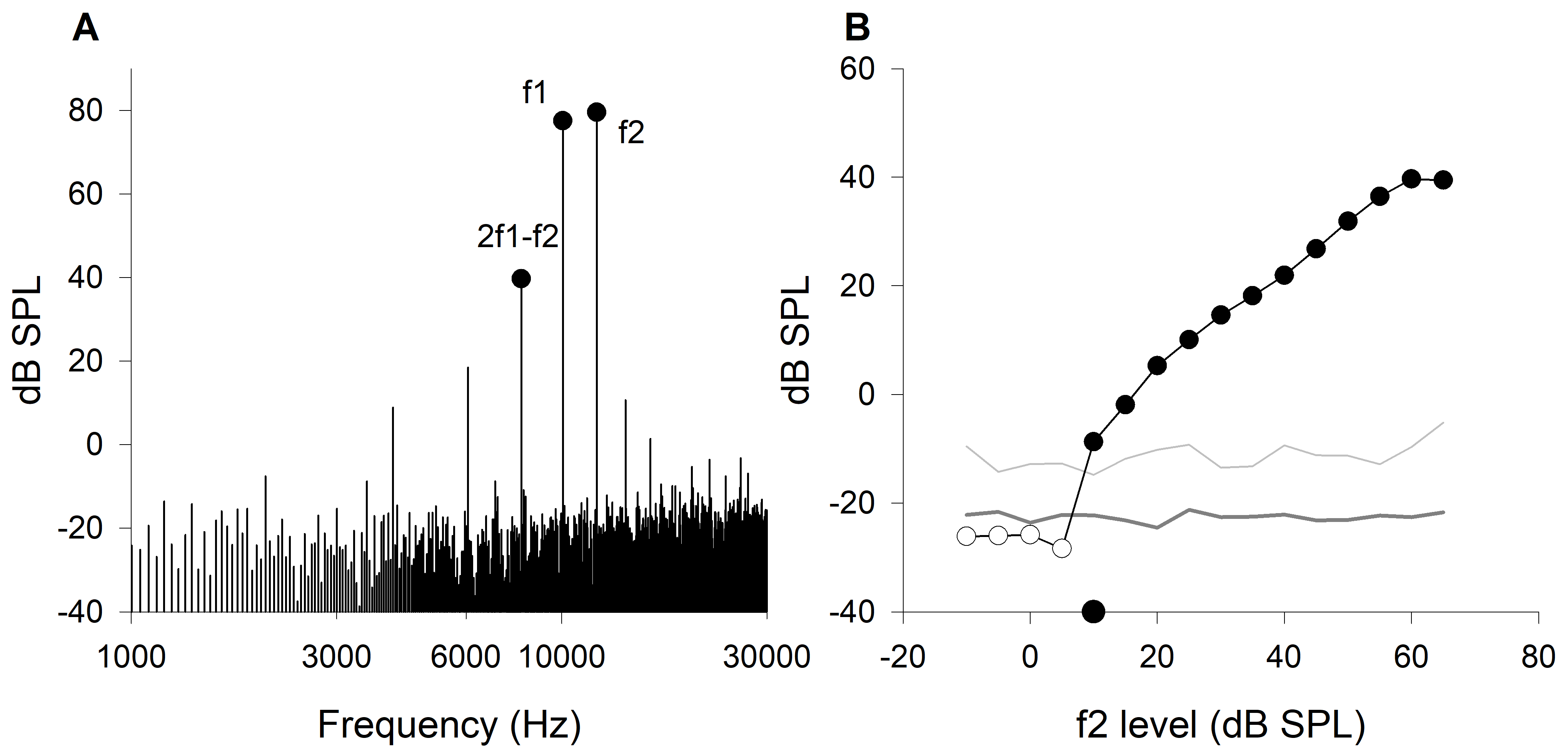

Supplement: S2 Fig — A. A representative DPOAE frequency spectrum is plotted for a response recorded with a stimulus of 2 frequency components, f1 (10,000 Hz, 70 dB SPL) and f2 (12,000 Hz, 60 dB SPL), for a wildtype Klhl18+/+ mouse. The spectral peaks of the two stimulus tones are labelled f1 and f2. The measured DPOAE is labelled 2f1-f2. These peaks are further highlighted by filled circles. Similar spectra were recorded using f2 levels from -10 dB to 65 dB and used to create a growth function for the 2f1-f2 DPOAE. B. For the same Klhl18+/+ mouse, DPOAE amplitude is plotted as a function of f2 level (circles). The thick grey line indicates the mean noise-floor amplitude for each response. The thin grey line indicates 2 standard deviations (SD) above the mean for the noise-floor. This line is used to estimate a threshold for the DPOAE component; the lowest f2 stimulus level where the DPOAE is greater than 2 SDs above the noise-floor. Sub-threshold DPOAEs are indicated by open circles. Supra-threshold DPOAEs are indicated by black filled circles. The estimated threshold is indicated by the single symbol on the abscissa. (TIF) [file pone.0258158.s002.tif]

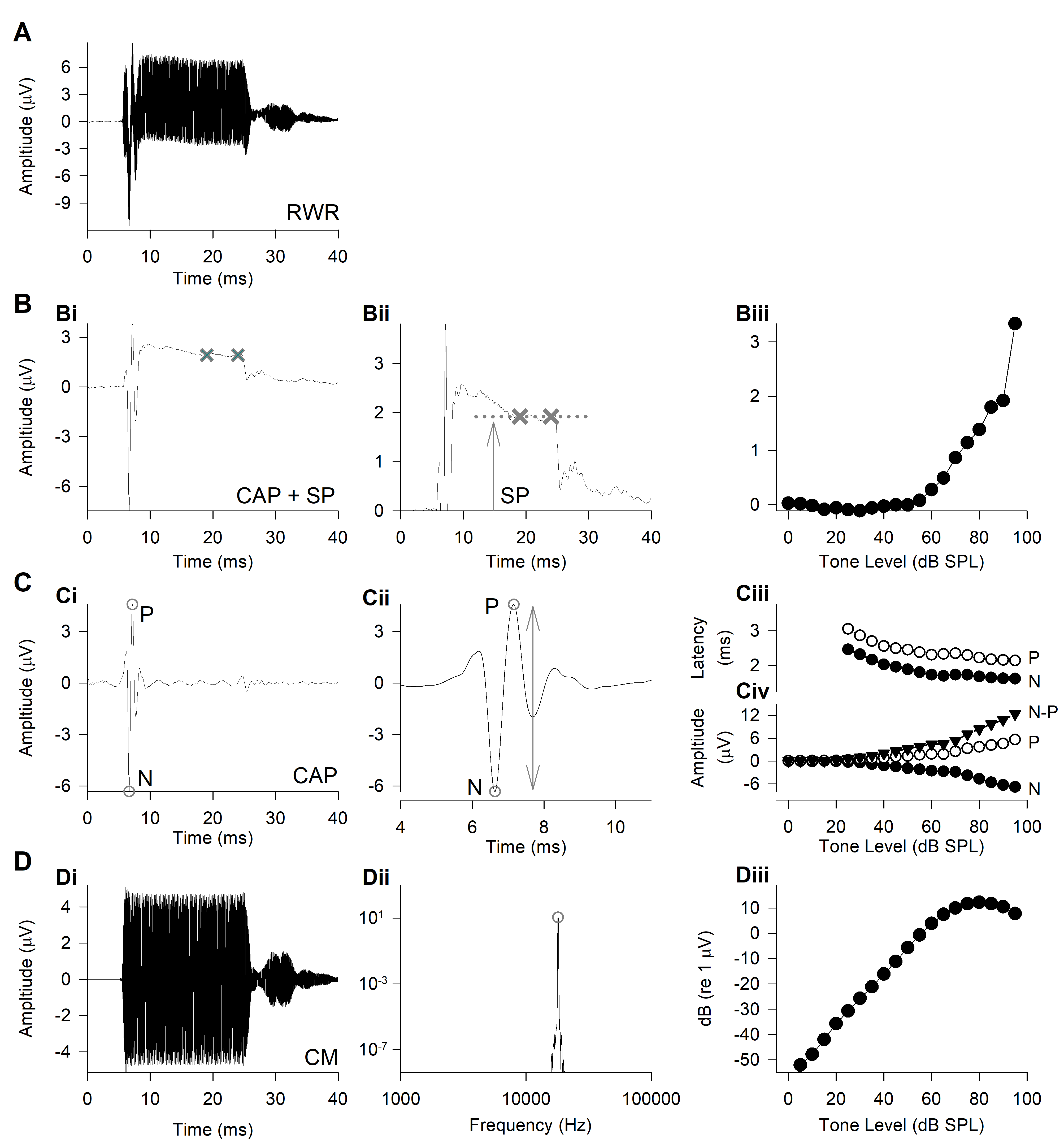

Supplement: S3 Fig — A. A typical electrophysiological response recorded from the mouse round window with a 1 Hz-50 kHz bandwidth bioamplifier; in this case, in response to an 18 kHz tone pip at 90 dB SPL. Bi. The response in (A), low pass filtered at 3 kHz to remove the cochlear microphonic (CM) component and expose the summating potential (SP) and cochlear compound action potential (CAP). The “X” symbols indicate points on the response at 19 ms and 24 ms. Data points between these time values were averaged to generate the magnitude of the SP. Bii illustrates that the SP is measured as a voltage magnitude displaced from the zero baseline of the lowpass filtered response. The SP can be positive or negative relative to the baseline. Biii. SP is plotted against dB SPL to produce a SP input-out function (IOF). Ci. The RWR from (A), bandpass filtered (300 Hz– 3 kHz) to remove both the CM and SP components and isolate the CAP response, composed of a large negative peak (N) followed by a large positive peak (P), indicated by open circle symbols. Cii illustrates the same filtered response from Ci but with the abscissa expanded to clearly show the structure of the CAP waveform. Peaks N and P are again illustrated by open circles. The arrow indicates the peak-to-peak (N to P) magnitude of the CAP. The latency of the N and P peaks was also measured (corrected to account for the 5ms onset delay of the stimulus tone pip). Ciii. The latency of peaks N and P (filled and open circles respectively) are plotted against dB SPL to produce a latency IOF. Civ. Similarly, the amplitude of peaks N and P and the N-P amplitude are plotted against dB SPL to produce amplitude IOFs. Di. The RWR from (A) bandpass filtered, centred on the stimulus frequency with high- and low- pass corner frequencies at ± 100 Hz, to remove all components within the response other than the CM. Dii. The filtered CM response is limited to a 7–23 ms time window before applying a Fast Fourier Transformation; the resulting power spectrum [file pone.0258158.s003.tif]

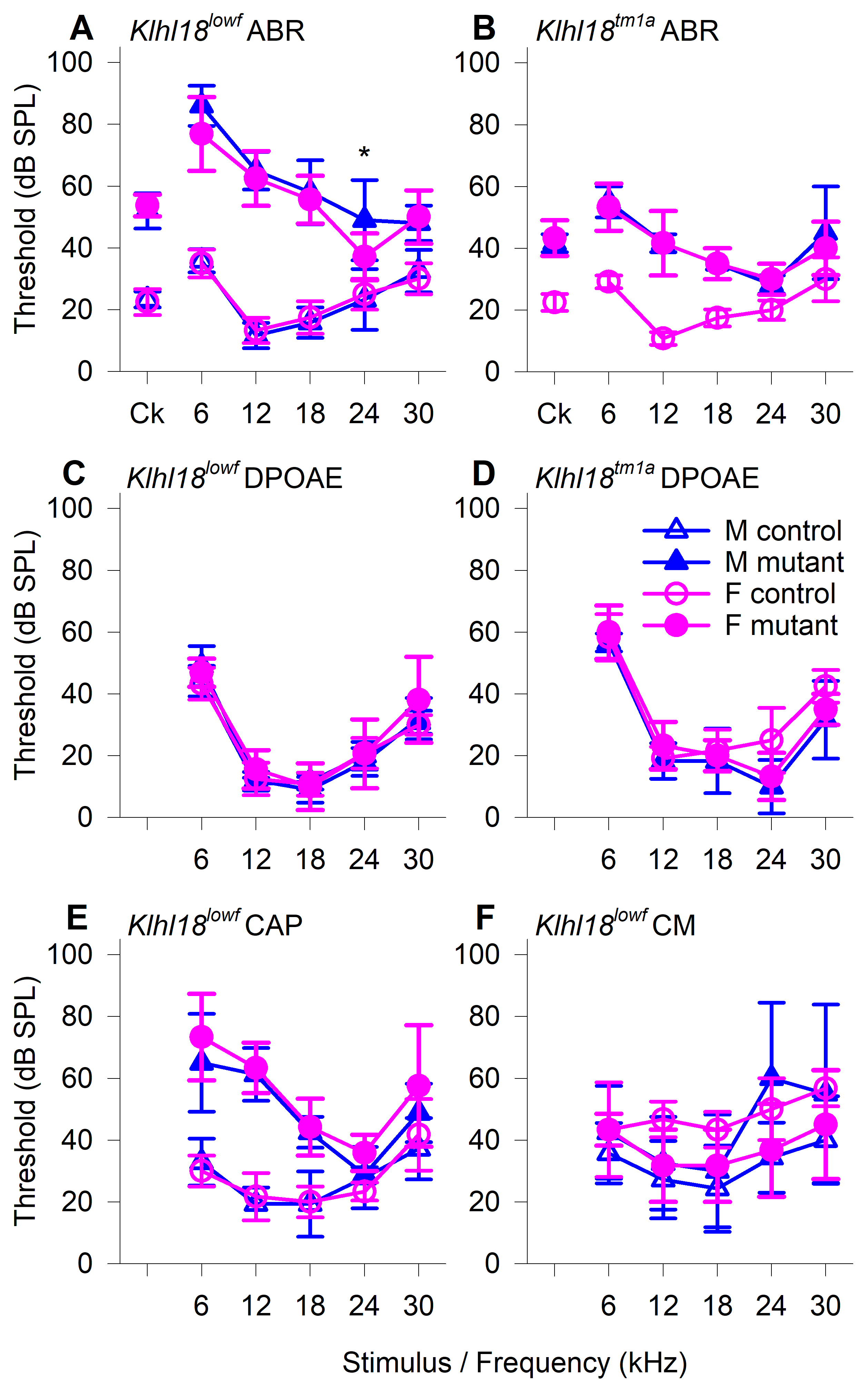

Supplement: S4 Fig — Mean (± SD) thresholds for male (blue triangles) and female (pink circles) Klhl18 control mice (open symbols) and Klhl18 mutant mice (filled symbols) are plotted in A ABR thresholds of Klhl18lowf mice (n = 6 control male, n = 6 control female, n = 5 mutant male and n = 8 mutant female), B ABR thresholds of Klhl18tm1a mice(n = 6 control female, n = 3 mutant male and n = 3 mutant female), C DPOAE thresholds of Klhl18lowf mice(n = 6 control male, n = 6 control female, n = 5 mutant male and n = 8 mutant female), D DPOAE thresholds of Klhl18tm1a mice(n = 6 control female, n = 3 mutant male and n = 3 mutant female), E CAP thresholds of Klhl18lowf mice (n = 7 wildtype male, n = 3 wildtype female, n = 4 mutant male and n = 6 mutant female) and F CM thresholds of Klhl18lowf mice (n = 7 wildtype male, n = 3 wildtype female, n = 4 mutant male and n = 6 mutant female). Where wildtype and heterozygote mice were combined to form a control group, these groups consisted of (A) 1 wildtype male, 5 heterozygote males, 2 wildtype females, 4 heterozygote females, (B) 1 wildtype female, 5 heterozygote females, (C) 1 wildtype male, 5 heterozygote males, 2 wildtype females, 4 heterozygote females and (D) 1 wildtype female, 5 heterozygote females. Results of a mixed-effects model statistical analysis between male and female control and mutant thresholds are shown in S1 Table. Sidak’s multiple comparisons test was used to examine the difference between control and mutant thresholds for each stimulus. Significant differences are indicated here by *. (TIF) [file pone.0258158.s004.tif]

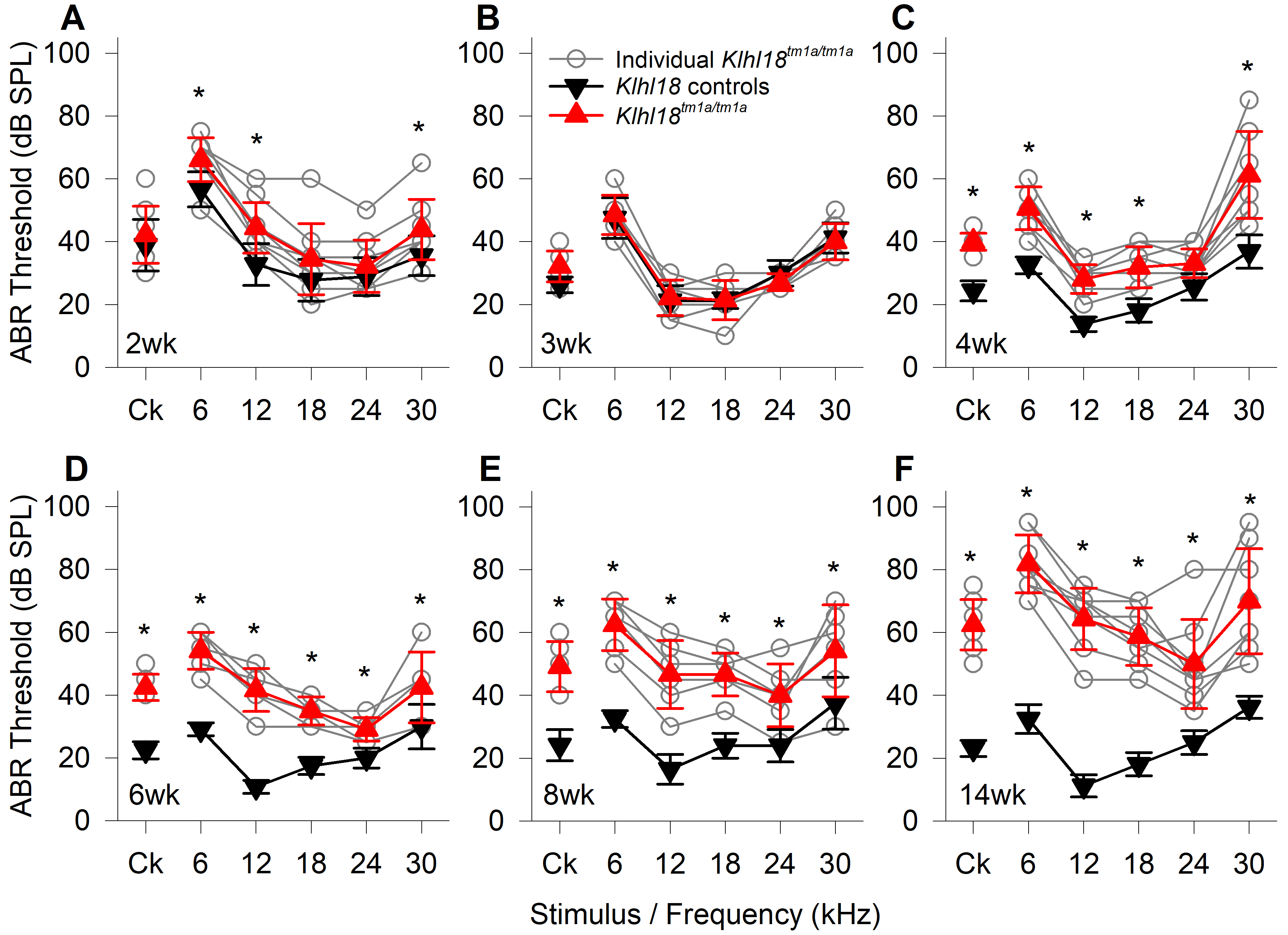

Supplement: S5 Fig — A summary of ABR thresholds estimated from control (wildtype or heterozygous) and mutant (homozygous) are shown for Klhl18tm1a mice aged 2 weeks (A, n = 9 control, including 3 wildtype, n = 9 mutant), 3 weeks (B, n = 4 control, all wildtype, n = 7 mutant), 4 weeks (C, n = 8 control, including 3 wildtype, n = 8 mutant), 6 weeks (D, n = 6 control, including 1 wildtype, n = 6 mutant), 8 weeks (E, n = 8 control, including 1 wildtype, n = 8 mutant) and 14 weeks (F, n = 8 control, including 3 wildtype, n = 8 mutant). Thresholds from individual mutant mice are indicated by grey circles. Mean threshold (±SD) are plotted for control mice as black down-triangles and for mutant mice as red up-triangles. Ck: Click stimulus. Results of a mixed-effects model statistical analysis between control and mutant thresholds are shown in S1 Table. Sidak’s multiple comparisons test was used to examine the difference between control and mutant thresholds for each stimulus. Significant differences are indicated here by *. (TIF) [file pone.0258158.s005.tif]

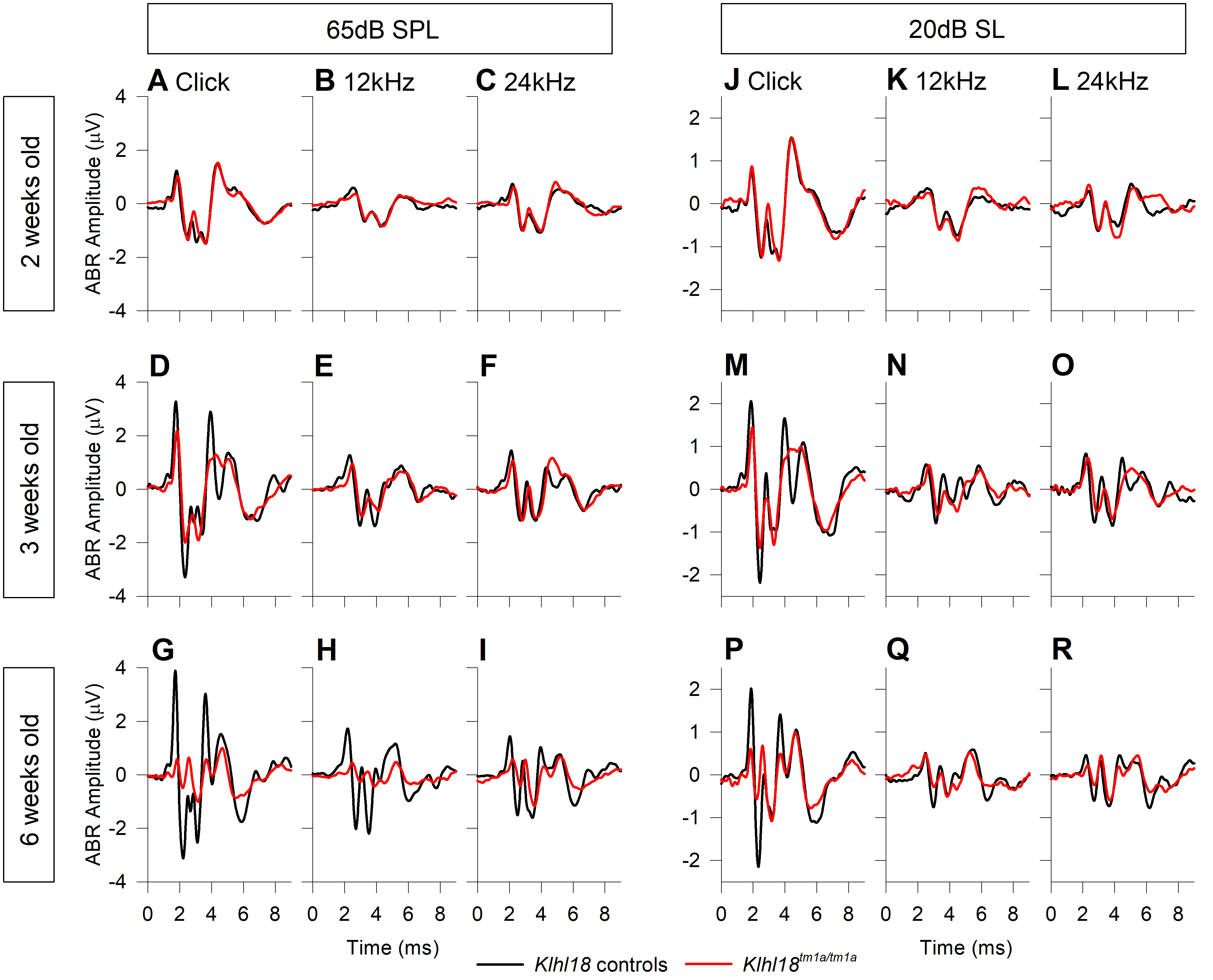

Supplement: S6 Fig — Group averaged ABR waveforms, evoked by 65 dB SPL stimuli, are plotted for mutant mice and heterozygous littermate controls aged 2 weeks (A-C), 3 weeks (D-F) and 6 weeks (G-I) in response to click stimuli (A,D,G), 12 kHz stimuli (B,E,H) and 24 kHz stimuli (C,F,I). Group averaged ABR waveforms, evoked by stimuli presented at 20 dB above threshold (20 dB sensation level, dB SL), are plotted for mice aged 2 weeks (J-L), 3 weeks (M-O), 6 weeks (P-R) in response to click stimuli (J,M,P), 12 kHz stimuli (K,N,Q) and 24 kHz stimuli (L,O,R). Group sizes were, at 2 weeks old, n = 9 control mice (n = 3 wildtypes and n = 6 heterozygotes), n = 9 mutants; at 3 weeks old, n = 4 control mice (all wildtypes), n = 7 mutants; at 6 weeks old, n = 6 controls (n = 1 wildtype and n = 5 heterozygotes), n = 6 mutants. Mean amplitude waveforms are plotted for the control mice (black lines) and mutant mice (red lines). (TIF) [file pone.0258158.s006.tif]

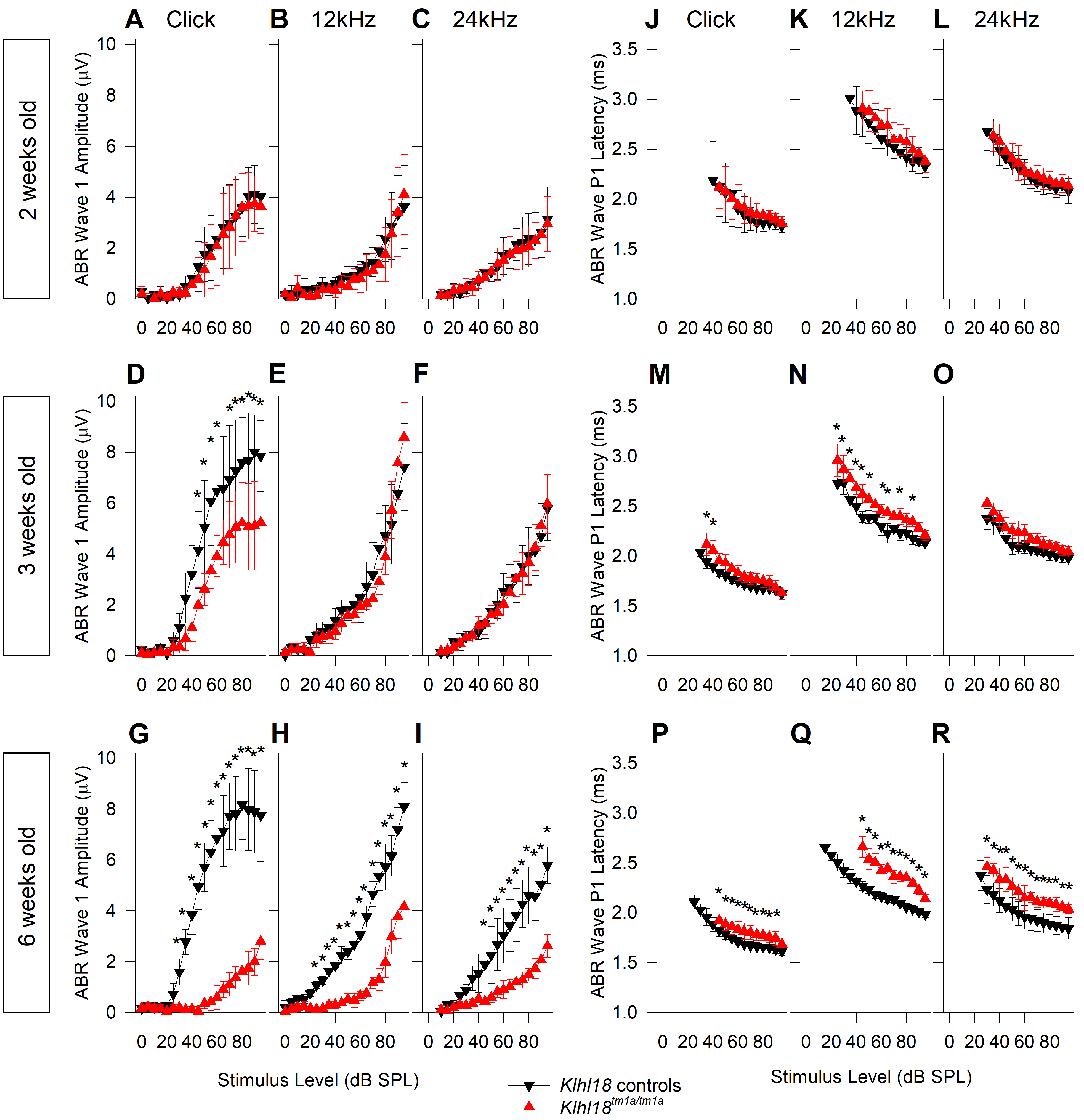

Supplement: S7 Fig — Mean ABR wave 1 P1-N1 peak-to-peak amplitude (±SD) as a function of stimulus level is plotted for mice aged 2, 3 and 6 weeks old are plotted in A-C (n = 9 controls, including 3 wildtypes; n = 9 mutants), D-F (n = 4 wildtype controls, n = 7 mutants) and G-I (n = 6 controls, including 1 wildtype; n = 6 mutants), respectively. Results are plotted for click stimuli (A,D,G), 12 kHz tones (B,E,H) and 24 kHz tones (C,F,I). Data from control mice are plotted as black down-triangles. Data from mutant mice are plotted as red up-triangles. Mean ABR wave P1 latency (±SD) as a function of stimulus level is plotted for the same mice aged 2, 3 and 6 weeks old are plotted in J-L, M-O and P-R, respectively. Results are plotted for click stimuli (J,M,P), 12 kHz tones (K,N,Q) and 24 kHz tones (L,O,R). Results of a mixed-effects model statistical analysis between control and mutant thresholds are shown in S1 Table. Sidak’s multiple comparisons test was used to examine the difference between control and mutant data for each stimulus. Significant differences are indicated here by *. (TIF) [file pone.0258158.s007.tif]

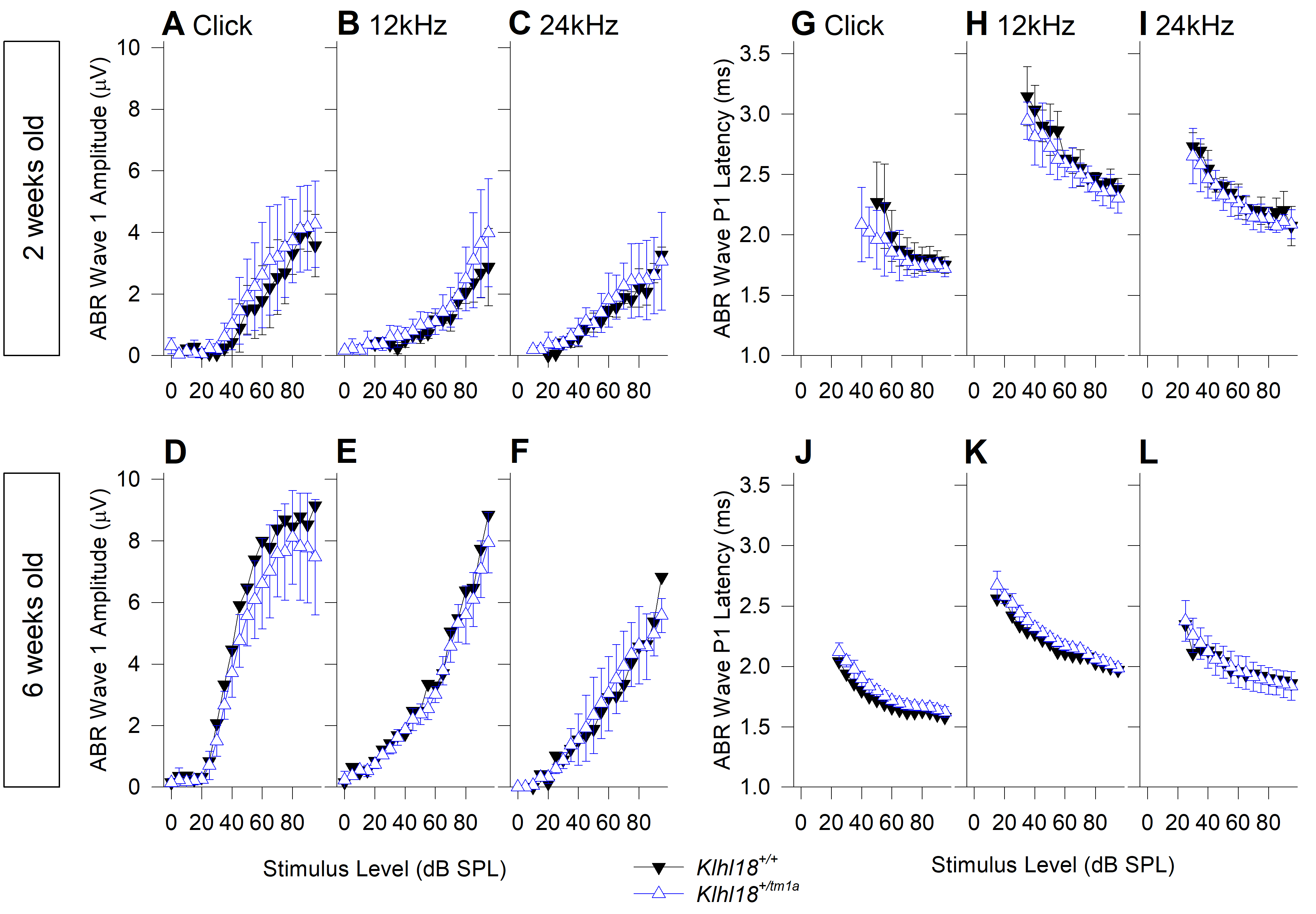

Supplement: S8 Fig — Mean ABR wave 1 P1-N1 peak-to-peak amplitude (±SD) as a function of stimulus level is plotted for mice aged 2 and 6 weeks old in A-C (n = 3 wildtype; n = 6 heterozygotes), and D-F (n = 1 wildtype; n = 5 heterozygotes), respectively. Results are plotted for click stimuli (A,D), 12 kHz tones (B,E) and 24 kHz tones (C,F). Data from wildtype mice are plotted as black down-triangles. Data from heterozygote mice are plotted as open blue up-triangles. Mean ABR wave P1 latency (±SD) as a function of stimulus level is plotted for the same mice aged 2 and 6 weeks old are plotted in G-I and J-L, respectively. Results are plotted for click stimuli (G,J), 12 kHz tones (H,K) and 24 kHz tones (I,L). Results of a mixed-effects model statistical analysis between control and mutant thresholds are shown in S1 Table. Sidak’s multiple comparisons test was used to examine the difference between wildtype and heterozygous mouse data for each stimulus. No significant differences were observed. (TIF) [file pone.0258158.s008.tif]

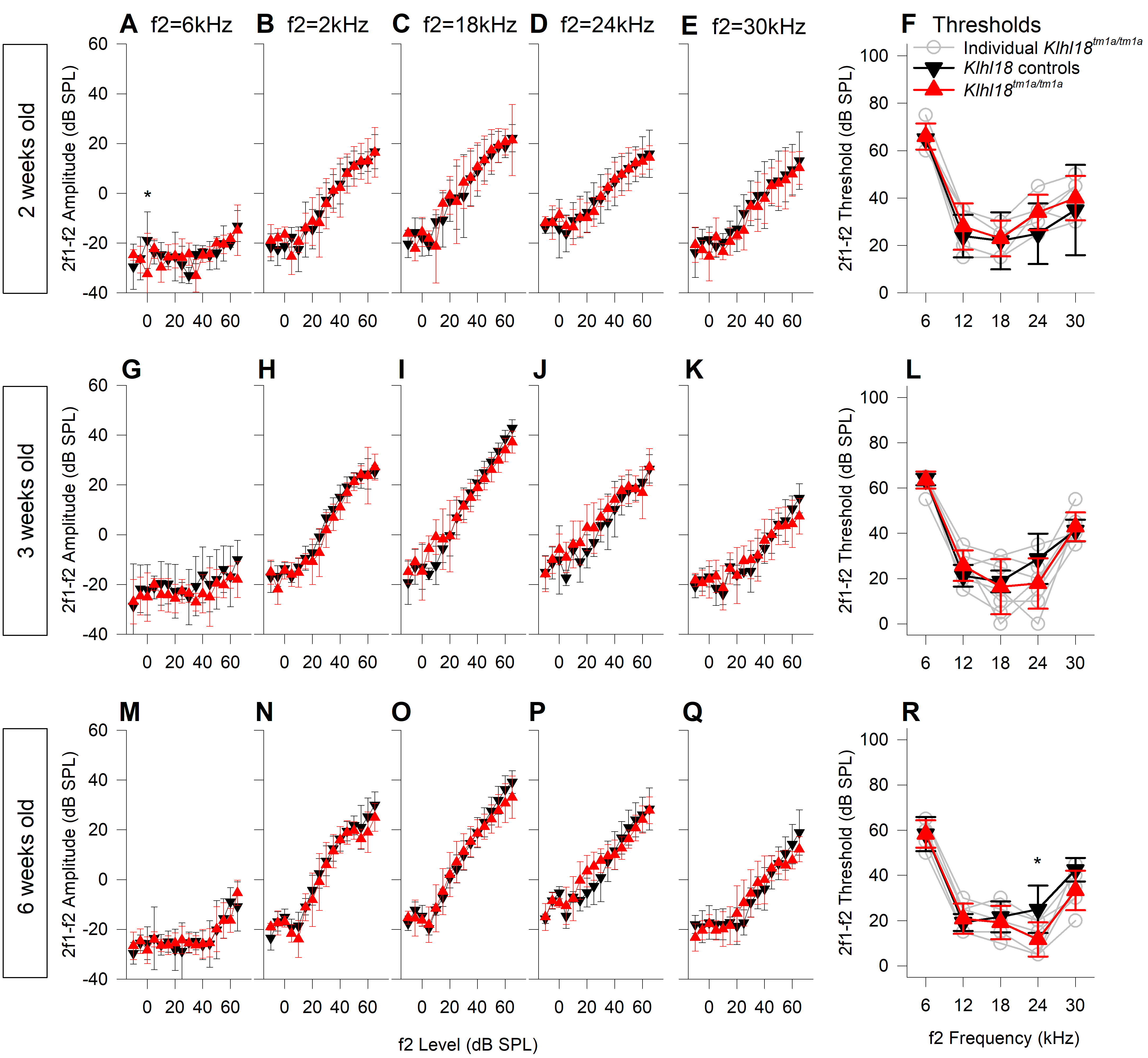

Supplement: S9 Fig — DPOAE results from mutant mice and littermate controls aged 2, 3 and 6 weeks old are plotted in A-F (n = 5 controls, comprised of 2 wildtypes and 3 heterozygotes; n = 5 mutants), G-L (n = 4 wildtype controls; n = 7 mutants) and M-N (n = 6 controls, comprised of 1 wildtype and 5 heterozygotes; n = 6 mutants), respectively. Data from control mice are plotted as black down-triangles. Data from mutant mice are plotted as red up-triangles. The mean (±SD) amplitude of the 2f1-f2 DPOAE (dB SPL) is plotted as a function of f2 stimulus level (dB SPL) for f2 frequencies of 6 kHz (A, G, M), 12 kHz (B, H, N), 18 kHz (C, I, O), 24 kHz (D, J, P) and 30 kHz (E, K, Q). Mean threshold (±SD) of the 2f1-f2 DPOAE (derived from individual growth functions, eg shown in S2 Fig) and plotted in F, L and R for mice aged 2 weeks, 3 weeks and 6 weeks respectively. In addition to the mean data, thresholds from individual mutant mice are plotted as grey open circles. Results of a mixed-effects model statistical analysis between control and mutant data are shown in S1 Table. Sidak’s multiple comparisons test was used to examine the difference between control and mutant data for each stimulus. Significant differences are indicated here by *. (TIF) [file pone.0258158.s009.tif]

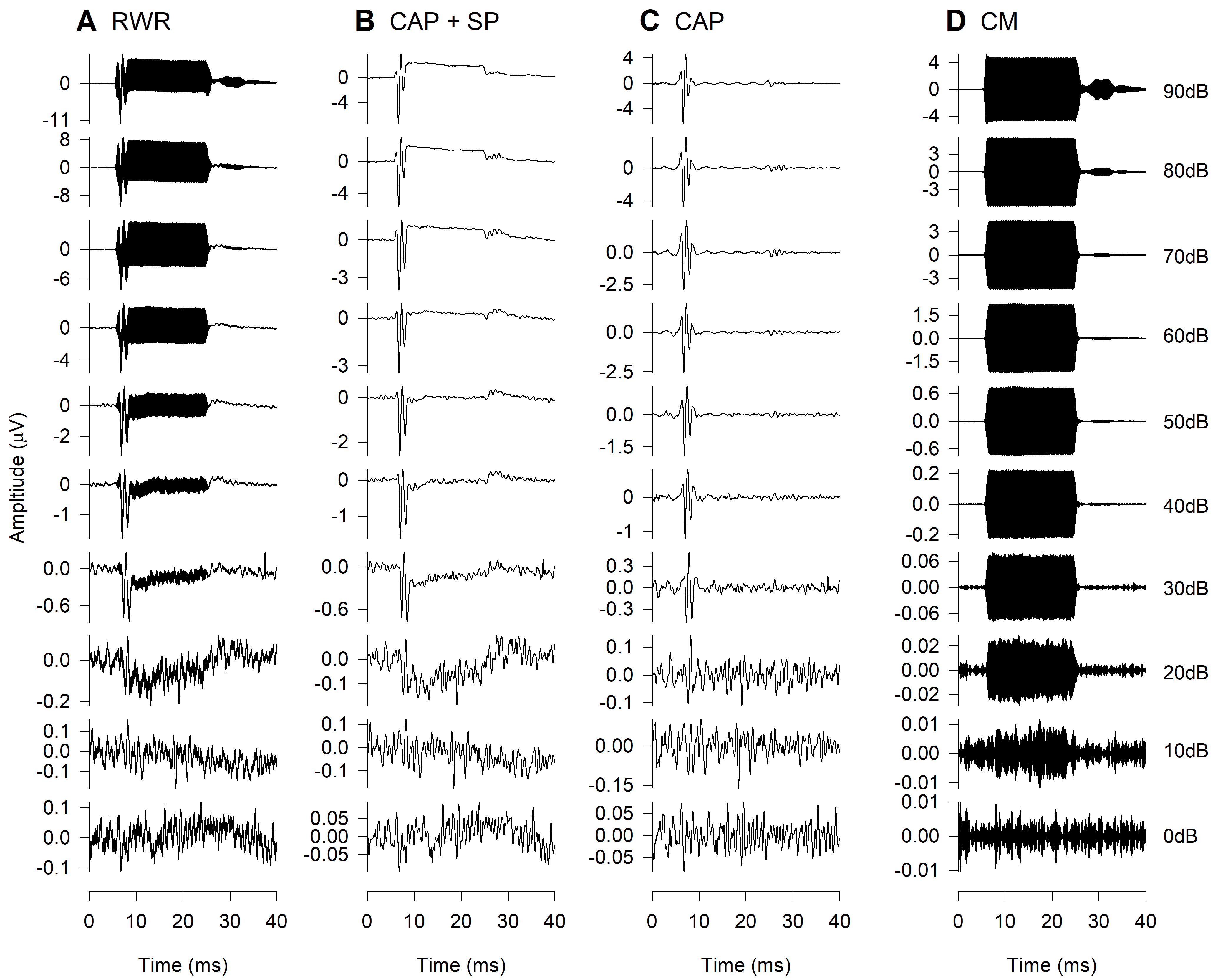

Supplement: S10 Fig — Representative responses recorded for 18 kHz tones in a control mouse, over stimulus levels from 0–90 dB SPL are illustrated. A. Broadband RWRs (1 Hz– 50 kHz). B. Lowpass filtered (3 kHz) RWRs show responses containing the CAP and SP. C. Bandpass filtered (300 Hz—3 kHz) RWRs show responses containing the CAPs. D. Bandpass filtered (18 kHz ± 100 Hz) RWRs show responses containing the CM. (TIF) [file pone.0258158.s010.tif]

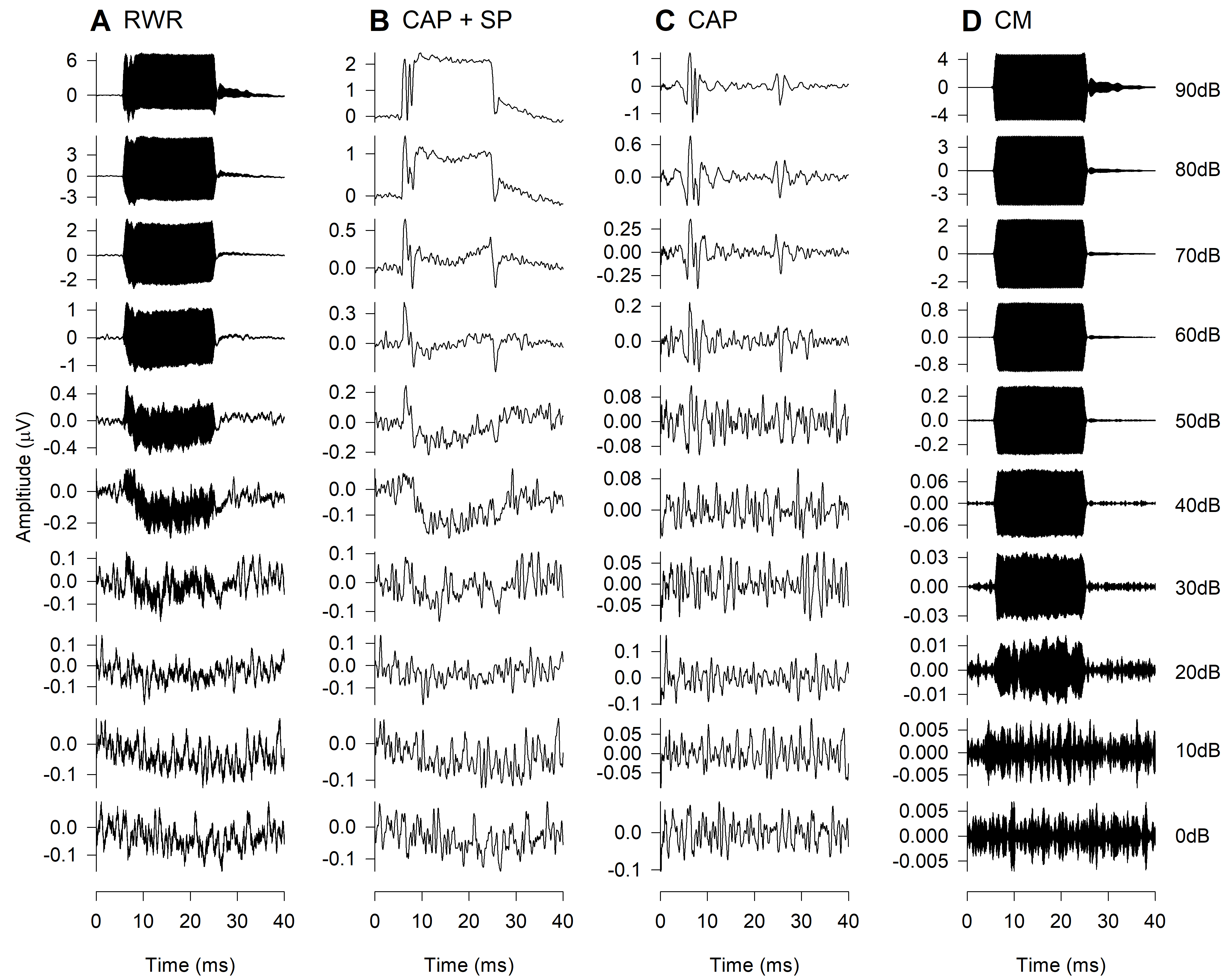

Supplement: S11 Fig — Representative responses recorded for 18kHz tones in a control mouse, over stimulus levels from 0–90 dB SPL are illustrated. A. Broadband RWRs (1 Hz– 50 kHz). B. Lowpass filtered (3 kHz) RWRs show responses containing the CAP and SP. C. Bandpass filtered (300 Hz– 3 kHz) RWRs show responses containing the CAPs. D. Bandpass filtered (18 kHz ± 100 Hz) RWRs show responses containing the CM. (TIF) [file pone.0258158.s011.tif]

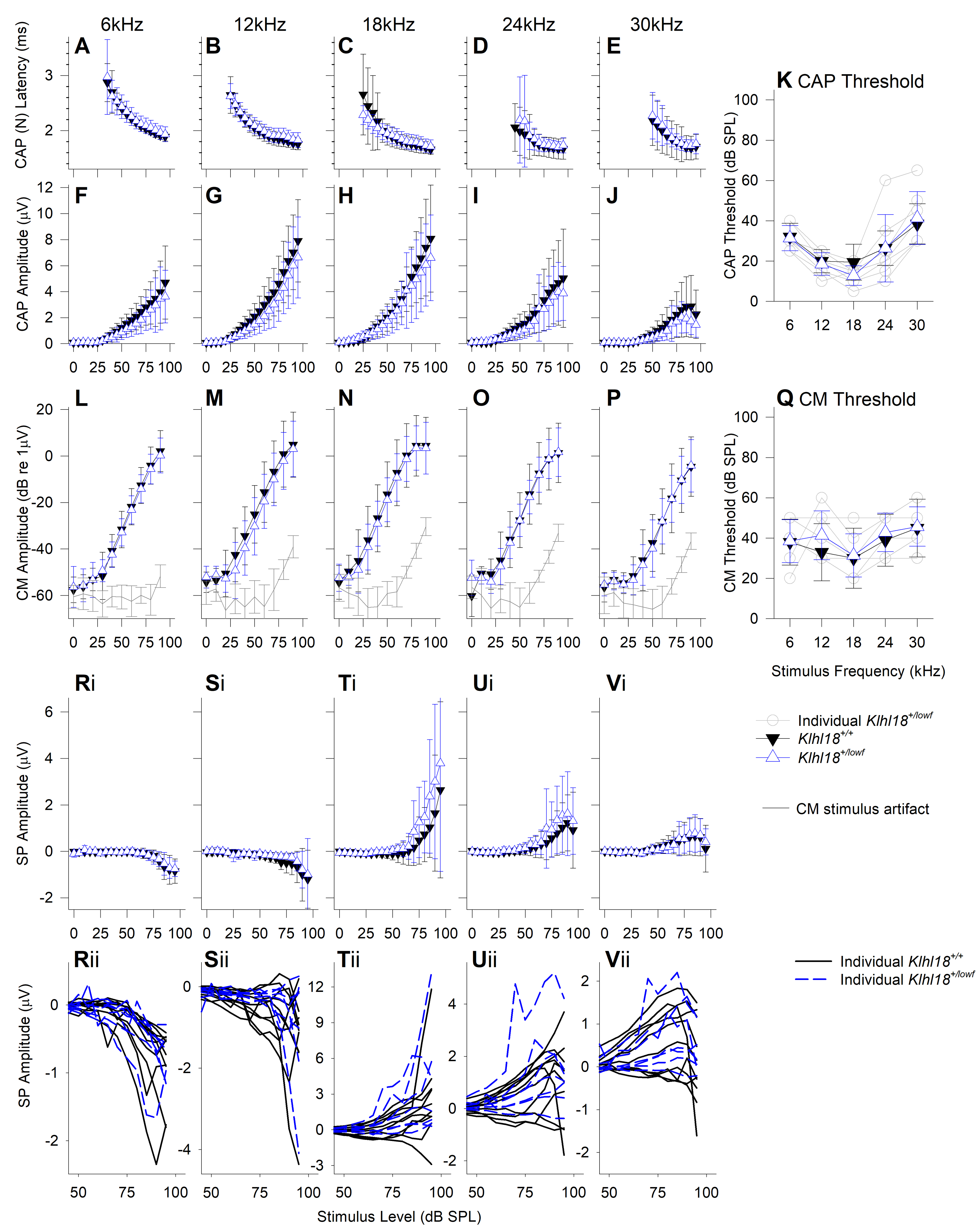

Supplement: S12 Fig — Compound Action Potential latency (of wave N) and amplitude (N-P amplitude) are plotted in A-E and F-J, respectively, for potentials measured in response to tones of 6, 12, 18, 24 and 30 kHz. Data are plotted as mean ± SD for wildtype control mice (n = 10; black down-triangles) and heterozygote mice (n = 7; blue up-triangles). K. Mean ± SD of the CAP threshold is plotted against stimulus frequency for wildtype control mice (black down-triangles) and heterozygote mice (blue up-triangles). Open circles and grey lines indicate CAP thresholds for individual heterozygote mice. L-P plot mean (± SD) Cochlear Microphonic amplitude (dB re 1 μV) against stimulus level (dB SPL) for wildtype mice (black down-triangles) and heterozygote mice (blue up-triangles). The line plotted in grey represents the mean ± SD amplitude of stimulus artifact. Q. Mean ± SD of the estimated CM threshold is plotted against stimulus frequency for wildtype control mice (black down-triangles) and heterozygote mice (blue up-triangles). Open circles and grey lines indicate CM thresholds for individual heterozygote mice. The grey line without symbols indicates the estimated magnitude of the stimulus artifact, as described in Fig 6. Ri-Vi plot mean (± SD) Summating Potential amplitude (μV) against stimulus level (dB SPL) for wildtype mice (black down-triangles) and heterozygote mice (blue up-triangles). Rii-Vii plot SP IOFs for individual control (black lines) and mutant (blue lines) mice. (TIF) [file pone.0258158.s012.tif]
