# Supplementary material for: Inner hair cell dysfunction in Klhl18 mutant mice leads to low frequency progressive hearing loss
Source: PLoS One. 2021 Oct 1;16(10):e0258158. doi: 10.1371/journal.pone.0258158 (PMC8486144; doi:10.1371/journal.pone.0258158)
Supplement: Attachment — Submitted filename: Ingham et al Klhl18 Response to Reviewers.docx [file pone.0258158.s014.docx]

**Responses to reviewers’ comments, Ingham et al.**

1. When submitting your revision, we need you to address these additional requirements. Please ensure that your manuscript meets PLOS ONE's style requirements, including those for file naming. The PLOS ONE style templates can be found at 
[https://journals.plos.org/plosone/s/file?id=wjVg/PLOSOne_formatting_sample_main_body.pdf](https://eur03.safelinks.protection.outlook.com/?url=https%3A%2F%2Fjournals.plos.org%2Fplosone%2Fs%2Ffile%3Fid%3DwjVg%2FPLOSOne_formatting_sample_main_body.pdf&data=04%7C01%7Ckaren.steel%40kcl.ac.uk%7C46e825f5ee0643acb0f108d96f81a378%7C8370cf1416f34c16b83c724071654356%7C0%7C0%7C637663424844793906%7CUnknown%7CTWFpbGZsb3d8eyJWIjoiMC4wLjAwMDAiLCJQIjoiV2luMzIiLCJBTiI6Ik1haWwiLCJXVCI6Mn0%3D%7C1000&sdata=6fGsLyoMNVmljEQpsp8LTS3XSrhuCtLz5hfF2bxirhs%3D&reserved=0) and 
[https://journals.plos.org/plosone/s/file?id=ba62/PLOSOne_formatting_sample_title_authors_affiliations.pdf](https://eur03.safelinks.protection.outlook.com/?url=https%3A%2F%2Fjournals.plos.org%2Fplosone%2Fs%2Ffile%3Fid%3Dba62%2FPLOSOne_formatting_sample_title_authors_affiliations.pdf&data=04%7C01%7Ckaren.steel%40kcl.ac.uk%7C46e825f5ee0643acb0f108d96f81a378%7C8370cf1416f34c16b83c724071654356%7C0%7C0%7C637663424844793906%7CUnknown%7CTWFpbGZsb3d8eyJWIjoiMC4wLjAwMDAiLCJQIjoiV2luMzIiLCJBTiI6Ik1haWwiLCJXVCI6Mn0%3D%7C1000&sdata=h5ZjmsgrP25ty%2BhOJ6WAwJDCKvA8BaTF1c5d61EExvo%3D&reserved=0)

We have edited the manuscript to comply with the journal formatting requirements and tracked all changes.

2. Please review your reference list to ensure that it is complete and correct. If you have cited papers that have been retracted, please include the rationale for doing so in the manuscript text, or remove these references and replace them with relevant current references. Any changes to the reference list should be mentioned in the rebuttal letter that accompanies your revised manuscript. If you need to cite a retracted article, indicate the article’s retracted status in the References list and also include a citation and full reference for the retraction notice.

We have checked the reference list but have not noticed any publications that we know have been retracted.

3. Please include a copy of Table S1 which you refer to in your text on page 13, 14, 16, 17, 18, 20, 21, 38, 39, 40 and 41.

We apologise for overlooking the inclusion of Table S1 in the original submission. An Excel file containing Table S1 (named “_TableS1_Ingham et al.xlsx”) is included with this revision.

Reviewer #1: The manuscript from Neil Ingham and co-authors entitled “Inner hair cell dysfunction in Klhl18 mutant mice leads to low frequency progressive hearing loss” is precisely crafted and has an abundance of supporting data that is presented in good-looking figures. It so nice at least once in a while to read a manuscript in near final form with thorough evaluations and properly replicated data.

Thank you for these comments.

There are only a few minor issues to suggest to the authors.

Klhl18lowf mutant mice had abnormally tapering inner hair cell stereocilia in the apical half of the cochlea while their synapses appeared normal.” The stereocilia taper usually refers to the base of a stereocilium where the stereocilium joins with the apical surface of the hair cell. That’s not the taper the authors are referring to and that becomes obvious when examining the beautiful images provided. The authors should make an effort to better define both in the abstract and in the main text that they are referring to abnormal tapers near the distal end of stereocilia.

Thank you for pointing out that confusion. We have altered the text at multiple locations to clarify that we are talking about tapering of the tips of stereocilia, not the insertion point into the cuticular plate.

The first time a variant of Klhl18 is mentioned, indicate if the allele is dominant or recessive.

We have added comments in the abstract and introduction that the hearing loss is recessive in inheritance. However, there may be a semi-dominant effect on stereocilia tip tapering. It is the phenotype studied that is recessive or dominant rather than the allele, so we are cautious in the text to describe hearing loss as recessive, not the full phenotype. We found no differences in our measures of auditory function between heterozygotes and wildtypes.

The statement on line 51 “Neither allele produced overt vestibular phenotypes and homozygotes are viable and fertile [1,2].” could be improved by mentioning the vestibular tests. “overt” isn’t very scientific. What vestibular abnormal phenotype would be classified as overt that these mice didn’t exhibit?

We have added “such as circling or head-bobbing” to the text at this point to clarify.

Reviewer #2: Inner hair cell dysfunction in Klhl18 mutant mice leads to low frequency progressive hearing loss

The manuscript deals with characterization of a model for a hearing phenotype that is found in, but less well studied, in the human population. The Klhl18 (Kelch-like family member 18) gene was examined using two different alleles in the mouse. Interestingly, this gene has been implicated in retina function. The morphological changes in the mice were subtle, with normal DPOAE, CM and synaptic contacts. However, the inner hair cells had some morphological changes (tapering in the inner hair cells), backed up by summating potentials, implicating these cells as impaired in the Klhl18 mutants. The analysis is extremely thorough, and provides a mechanism of function for the low frequency progressive hearing loss due to mutations in this gene. It will be interesting to see if human pathogenic variants might turn up if such families will be screened by whole exome sequencing.

Thank you for these comments. We are looking out for human mutations as the reviewer suggests.
